# Quantifying the roles of random motility and directed motility using advection-diffusion theory for a 3T3 fibroblast cell migration assay stimulated with an electric field

**DOI:** 10.1186/s12918-017-0413-5

**Published:** 2017-03-17

**Authors:** Matthew J. Simpson, Kai-Yin Lo, Yung-Shin Sun

**Affiliations:** 1 School of Mathematical Sciences, Queensland University of Technology (QUT), Brisbane, Australia; 2 Department of Agricultural Chemistry, National Taiwan University, Taipei, 10617 Taiwan; 3 Department of Physics, Fu-Jen Catholic University, New Taipei City, 24205 Taiwan

**Keywords:** Cell migration, Random motility, Directed motility, Electrotaxis, Partial differential equation, Keller-Segal model

## Abstract

**Background:**

Directed cell migration can be driven by a range of external stimuli, such as spatial gradients of: chemical signals (chemotaxis); adhesion sites (haptotaxis); or temperature (thermotaxis). Continuum models of cell migration typically include a diffusion term to capture the undirected component of cell motility and an advection term to capture the directed component of cell motility. However, there is no consensus in the literature about the form that the advection term takes. Some theoretical studies suggest that the advection term ought to include receptor saturation effects. However, others adopt a much simpler constant coefficient. One of the limitations of including receptor saturation effects is that it introduces several additional unknown parameters into the model. Therefore, a relevant research question is to investigate whether directed cell migration is best described by a simple constant tactic coefficient or a more complicated model incorporating saturation effects.

**Results:**

We study directed cell migration using an experimental device in which the directed component of the cell motility is driven by a spatial gradient of electric potential, which is known as electrotaxis. The electric field (*EF*) is proportional to the spatial gradient of the electric potential. The spatial variation of electric potential across the experimental device varies in such a way that there are several subregions on the device in which the *EF* takes on different values that are approximately constant within those subregions. We use cell trajectory data to quantify the motion of 3T3 fibroblast cells at different locations on the device to examine how different values of the *EF* influences cell motility. The undirected (random) motility of the cells is quantified in terms of the cell diffusivity, *D*, and the directed motility is quantified in terms of a cell drift velocity, *v*. Estimates *D* and *v* are obtained under a range of four different *EF* conditions, which correspond to normal physiological conditions. Our results suggest that there is no anisotropy in *D*, and that *D* appears to be approximately independent of the *EF* and the electric potential. The drift velocity increases approximately linearly with the *EF*, suggesting that the simplest linear advection term, with no additional saturation parameters, provides a good explanation of these physiologically relevant data.

**Conclusions:**

We find that the simplest linear advection term in a continuum model of directed cell motility is sufficient to describe a range of different electrotaxis experiments for 3T3 fibroblast cells subject to normal physiological values of the electric field. This is useful information because alternative models that include saturation effects involve additional parameters that need to be estimated before a partial differential equation model can be applied to interpret or predict a cell migration experiment.

**Electronic supplementary material:**

The online version of this article (doi:10.1186/s12918-017-0413-5) contains supplementary material, which is available to authorized users.

## Background

Continuum models are used to describe cell migration in a number of contexts including wound repair [1–3] and malignant invasion [4, 5]. Here, we consider a continuum partial differential equation to describe the motion of a population of cells, with cell density *C*(*x,y,t*), where *x* and *y* are the Cartesian coordinates, and *t* is time. The continuum model allows the cell migration mechanism to involve an undirected (diffusive) and directed (tactic) component. Conservation arguments lead to

1 $$ \begin{aligned} \frac{\partial{C}}{\partial{t}} &= \frac{\partial}{\partial{x}} \left(D(S)\frac{\partial{C}}{\partial{x}} \right)+ \frac{\partial}{\partial{y}} \left(D(S)\frac{\partial{C}}{\partial{y}} \right) \\ &- \frac{\partial}{\partial{x}}\left(\chi(S) \frac{\partial{S}}{\partial{x}} C \right) -\frac{\partial}{\partial {y}}\left(\chi(S) \frac{\partial{S}}{\partial{y}} C \right), \end{aligned}  $$

where *D*(*S*)>0 is the cell diffusivity, and *χ*(*S*) is the tactic sensitivity function. In this Keller-Segel [6] type model, the tactic flux is proportional to the gradient of some signal, *S*(*x,y,t*), and the strength of the tactic response is governed by the tactic sensitivity function, *χ*(*S*) [6, 7]. Setting *χ*(*S*)>0 represents attraction, since the directed component of the cell flux is in the direction of increasing *S*. Alternatively, setting *χ*(*S*)<0 represents repulsion. To maintain generality, the cell diffusivity *D*(*S*)>0 is also written as a function of the signal, *S* [1, 8, 9]. If *D*(*S*) is increasing, this model represents an increase in undirected motility with the signal, as in the case of chemokinesis [10]. Since there is no source/sink term in Eq. (1) we are focusing on cell migration processes on short time scales so that cell proliferation and cell death have a negligible impact on the cell density.

Directed cell migration can occur in response to various types of external spatial gradients. In Eq. (1) we have not specified the physical interpretation of *S*. In a model of chemotaxis *S* would represent the concentration of a chemical signal, whereas in a model of thermotaxis *S* would represent the temperature. In a model of electrotaxis *S* represents the electric potential. In this work we focus on stimulating directed cell migration in an electric field.

Electrotaxis plays an important role in guiding epithelial and corneal wound healing processes, and could potentially be used to design novel therapies [11–16]. While the precise molecular-level mechanisms behind electrotaxis remain unresolved, a common hypothesis is that exposing cells to an electric field leads to changes in plasma membrane potentials [11, 12] with the membrane facing the cathode becoming depolarized, and the membrane facing the anode becoming hyperpolarized [11, 12]. In a cell with negligible voltage-gated conductance, the hyperpolarized membrane attracts calcium ions, leading to a contraction of this side of the cell which propels the cell toward the cathode [11, 12]. In a cell with voltage-gated calcium channels, the channels near the depolarized side open to allow an influx of calcium ions leading to a rise in the intracellular calcium ion level throughout such a cell. The direction of cell movement in this situation will depend on the balance between the opposing contractile forces [11, 12].

A key question in applying Eq. (1) is to determine the functional forms of *D*(*S*) and *χ*(*S*). In many theoretical studies focusing on directed cell movement, an explicit relationship between the tactic response function and the signal, *S*, is emphasized. Often, particularly in more theoretical studies, an argument about saturation of receptor cites on cells is made to suggest that *χ*(*S*) ought to be a decreasing function of *S*, so that d*χ*/d*S*<0 [6, 7]. Several putative functional forms have been put forward. For example, relationships such as *χ*(*S*) = *χ*_0_/*S* and *χ*(*S*) = *χ*_0_ *K* / (*K*+*S*)^2^, and several others, have been suggested [13, 18–23]. In contrast, other studies simply adopt a constant *χ*(*S*)=*χ* [24–33]. Under the situation where we treat *D*(*S*) and *χ*(*S*) as constants, Eq. (1) simplifies to

2 $$\begin{array}{*{20}l} {}\frac{\partial{C}}{\partial{t}} &= D\left[\frac{\partial^{2}C}{\partial x^{2}} + \frac{\partial^{2} {C}}{\partial y^{2}} \right] - \chi \left[\frac{\partial}{\partial{x}}\left(\frac{\partial{S}}{\partial{x}} C \right) + \frac{\partial}{\partial{y}}\left(\frac{\partial{S}}{\partial{y}} C \right) \right]\!. \end{array} $$

The advantage of working with Eq. (2) compared to Eq. (1), is that there are just two unknown parameters in Eq. (2), *χ* and *D*. In contrast, the more complicated models involving receptor saturation effects can involve six or more unknown parameters [13, 18–23].

Making a distinction between choosing models where the tactic sensitivity incorporates receptor saturation effect (Eq. (1)) and a simpler model where the tactic sensitivity coefficient is constant (Eq. (2)) is not obvious unless we are guided by a reasonable quantity of experimental data. From a theoretical point of view, it might be attractive to incorporate receptor saturation dynamics into a mathematical model, but this comes with the trade off that this is typically achieved by introducing a complicated relationship between the tactic sensitivity coefficient and the attractant concentration, which can introduce several unknown parameters into the mathematical model thereby over complicating the process of model calibration [17]. To provide some insight into this question, here we analyze a suite of cell migration data. The data we analyze comes from an electrotaxis experiment where the strength of the attraction gradient is carefully varied so that we can analyze both the random component of the cell migration as well as the directed component over a range of applied gradients.

## Results

### Qualitative assessment of trajectory data

**Fig. 1:**
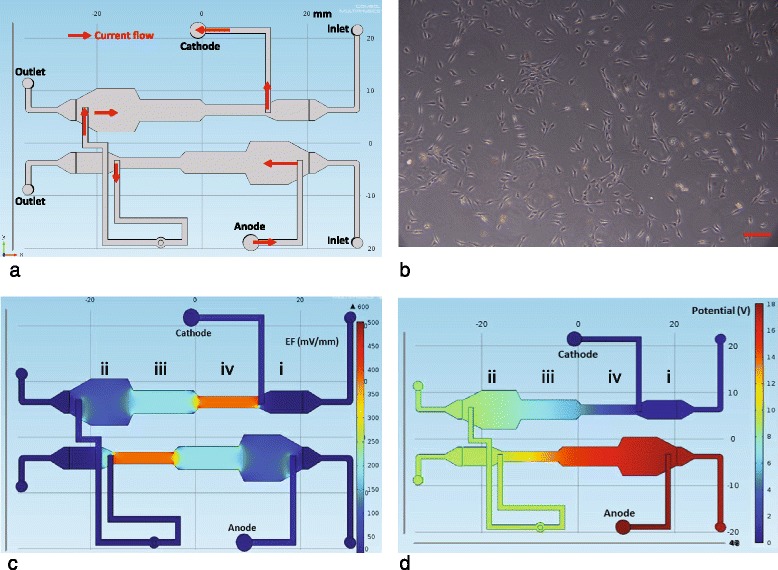
**a** Schematic of the microfluidic device indicating the direction of current flow, which corresponds to the direction in which cell migration is biased. **b** Image of 3T3 fibroblast cells, distributed at low density, during a typical experiment. The *scale bar* corresponds to 100 *μ*m. **c** COMSOL (COMSOL, USA) simulation showing the spatial distribution of the electric field *EF* (mV/mm) on the microfluidic device. *Markers* *i*, *ii*, *iii* and *iv* are placed on the approximate location where *EF*=0,100,200 and 400 mV/mm, respectively. These *markers* indicate the approximate location where the cell migration, for each value of *EF*, is observed. **d** COMSOL (COMSOL, USA) simulation of the spatial distribution of the potential (V) on the microfluidic device

Cell trajectory data, describing the motion of 80 randomly-chosen 3T3 fibroblast cells [34] (Fig. 1 b) under a range of gradients, *EF*=0,100,200 and 400 mV/mm, within the experimental apparatus (Fig. 1 a, c-d) are analysed [35]. Since 3T3 fibroblast cells are known to migrate towards the cathode in these types of experiments [35], the Cartesian coordinate axes are aligned so that the positive *x*-direction points towards the cathode (Fig. 1 c-d). We note that there is no gradient in the *y*-direction (Fig. 1 c-d).

**Fig. 2:**
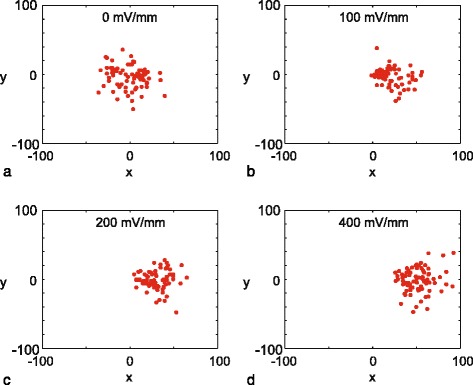
End points of cell trajectories under different experimental conditions. Results correspond to: **a** *EF*=0; **b** *EF*=100; **c** *EF*=200, and **d** *EF*=400 mV/mm. All trajectories are shifted so that the initial location of the trajectory is at the origin. In each subfigure there are 80 *red dots*, each corresponding to the location of the each cell after a duration of two hours

The data involves recording the initial position of each trajectory, (*x* ^′^(0),*y* ^′^(0)) and the position of each cell every half-hour over a two hour interval, giving: (*x* ^′^(0.5),*y* ^′^(0.5)), (*x* ^′^(1),*y* ^′^(1)), (*x* ^′^(1.5),*y* ^′^(1.5)) and (*x* ^′^(2),*y* ^′^(2)). Using this data, we shift the coordinate system for each trajectory so that the initial location of the cell is at the origin, giving (*x*(*t*),*y*(*t*))=(*x* ^′^(*t*)−*x* ^′^(0),*y* ^′^(*t*)−*y* ^′^(0)). Plots showing (*x*(2),*y*(2)) for 80 trajectories under four different gradients are shown in Fig. 2. The scatter plot in Fig. 2 a, under the action of no gradient, shows an approximately symmetric distribution of the end points of the trajectories. In this case the trajectories extend no further than approximately 40 *μ*m away from the origin. Since these trajectories appear to follow no particular preferred direction, this cells seem to undergo an unbiased migration process. In comparison, the scatter plot in Fig. 2 b shows that there is some drift in the positive *x*-direction when the cells move under the action of a gradient. Despite the fact that there is an obvious drift in the positive *x*-direction in Fig. 2 b, there remains some randomness in the distribution of (*x*(2),*y*(2)). Therefore, under the action of the electric field, these 3T3 fibroblast cells move with both a directed and an undirected component. Comparing results in Fig. 2 b-d confirms that the drift in the positive *x*-direction increases with the increasing electric field, and there appears to be some randomness in the distribution of cells regardless of the strength of the electric field. To provide more information about the roles of directed and undirected motion in these experiments, we will now interpret this data using a biased random walk model that is related to an advection-diffusion equation.

### Quantitative assessment of trajectory data

**Fig. 3:**
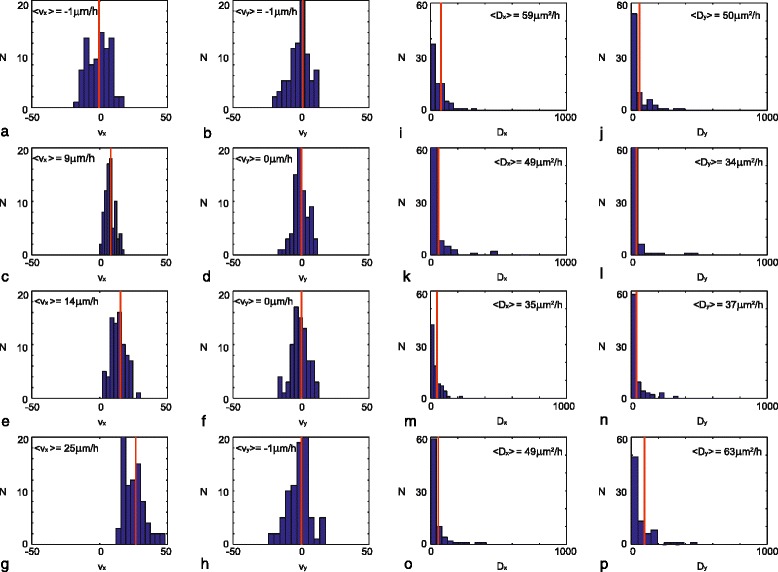
Histograms showing: **a** *v*_*x*_ for *EF*=0 mV/mm; **b** *v*_*y*_ for *EF*=0 mV/mm; **c** *v*_*x*_ for *EF*=100 mV/mm; **d** *v*_*y*_ for *EF*=100 mV/mm; **e** *v*_*x*_ for *EF*=200 mV/mm; **f** *v*_*y*_ for *EF*=200 mV/mm; **g** *v*_*x*_ for *EF*=400 mV/mm; **h** *v*_*y*_ for *EF*=400 mV/mm; **i** *D*_*x*_ for *EF*=0 mV/mm; **j** *D*_*y*_ for *EF*=0 mV/mm; **k** *D*_*x*_ for *EF*=100 mV/mm; **l** *D*_*y*_ for *EF*=100 mV/mm; **m** *D*_*x*_ for *EF*=200 mV/mm; **n** *D*_*y*_ for *EF*=200 mV/mm; **o** *D*_*x*_ for *EF*=400 mV/mm; **p** *D*_*y*_ for *EF*=400 mV/mm. A *red vertical line* is superimposed on each histogram to indicate the sample mean. The sample mean value for each histogram is indicated in the *top left* of each subfigure

We first quantify the directed component of the motility depicted in Fig. 2. Estimates of the drift velocity are obtained, in both the *x* and *y* directions, for each of the 80 trajectories, under the four different gradient conditions. These data are presented as histograms in Fig. 3. Results in Fig. 3 a-b characterize the estimates of *v*_*x*_ and *v*_*y*_ when there is no gradient, and averaging these 80 estimates gives us an approximation of the average drift velocity in each direction. This gives 〈*v*_*x*_〉=−1*μ*m/h and 〈*v*_*y*_〉=−1*μ*m/h. Therefore, the average drift velocity in both directions is approximately zero, as we anticipate intuitively by inspecting the data in Fig. 2 a. Results in Fig. 3 c-h show estimates of *v*_*x*_ and *v*_*y*_ for *EF*=100,200 and 400 mV/mm, respectively. In each case we see that 〈*v*_*y*_〉≈0*μ*m/h, which is consistent with the experimental design since there is no gradient in the *y* direction (Fig. 1 c-d). In contrast, estimates of 〈*v*_*x*_〉 increase with *EF*, as we have 〈*v*_*x*_〉=−1,9,14 and 25 *μ*m/h when *EF*=0,100,200 and 400 mV/mm, respectively. In addition to characterizing the mean drift velocities, 〈*v*_*x*_〉 and 〈*v*_*y*_〉, the data in the histograms in Fig. 3 a-h show how the individual estimates of *v*_*x*_ and *v*_*y*_ are distributed for each of the 80 trajectories considered. A qualitative assessment of these distributions indicates that, for each value of the *EF*, estimates of *v*_*x*_ and *v*_*y*_ are approximately symmetrically distributed about the mean. Furthermore, the spread about the mean appears to be approximately constant for each value of the *EF*.

Given our estimates of 〈*v*_*x*_〉 and 〈*v*_*y*_〉 (Fig. 3 a-h), we now estimate the diffusivity coefficients, *D*_*x*_ and *D*_*y*_, for each experiment. Results showing estimates of *D*_*x*_ and *D*_*y*_ under the application of no gradient are summarised in Fig. 3 i-j. Averaging our estimates across the 80 trajectories we obtain 〈*D*_*x*_〉=59 *μ*m^2^/h and 〈*D*_*y*_〉=50*μ*m^2^/h for the experiments in which there is no gradient. The magnitude of these estimates of cell diffusivity are consistent with previous estimates 3T3 fibroblast cells obtained using single cell trajectory data [36, 37]. Additional estimates of *D*_*x*_ and *D*_*y*_, and 〈*D*_*x*_〉 and 〈*D*_*y*_〉 are shown in Fig. 3 k-p for cell migration under the influence of gradients of 100, 200 and 400 mV/mm, respectively. For each of these data sets we have 〈*D*_*x*_〉≈〈*D*_*y*_〉, indicating that the random motility coefficient is isotropic. Furthermore, unlike our estimates of 〈*v*_*x*_〉, our estimates of 〈*D*_*x*_〉 and 〈*D*_*y*_〉 appear not to depend on the electric field.

### Relationship between the applied gradient, cell diffusivities and drift velocities

**Fig. 4:**
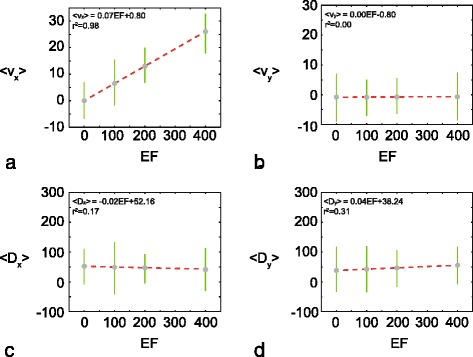
Summary of the average transport coefficients as a function of the applied gradient. **a** 〈*v*_*x*_〉 as a function of *EF*; **b** 〈*v*_*y*_〉 as a function of *EF*; **c** 〈*D*_*x*_〉 as a function of *EF*; and **d** 〈*D*_*y*_〉 as a function of *EF*. In each *plot* the sample mean is shown (*grey circle*), and the *error bars* indicate the variability. In this case the variability is reported as the one sample standard deviation about the mean. In each case an unconstrained linear regression is superimposed in each subfigure

To further explore the relationships between *D*_*x*_, *D*_*y*_, *v*_*x*_, *v*_*y*_ and the applied gradient, we calculate the sample mean and sample standard deviation for each of the 16 histograms in Fig. 3. Results in Fig. 4 show 〈*v*_*x*_〉, 〈*v*_*y*_〉, 〈*D*_*x*_〉 and 〈*D*_*y*_〉, each plotted as a function of the electric field. The plots show the variation in the average transport coefficients with the *EF*. In addition, the variability in the estimates of the average transport coefficients is indicated by the error bars. The error bars indicate the sample mean plus or minus one sample standard deviation.

Results in Fig. 4 a-b show 〈*v*_*x*_〉 and 〈*v*_*y*_〉 as a function of the *EF*. As we anticipate, 〈*v*_*x*_〉 increases with *EF* whereas 〈*v*_*y*_〉≈0 for all *EF* considered. To examine the putative relationship between 〈*v*_*x*_〉 and *EF*, and between 〈*v*_*y*_〉 and *EF*, we perform an unconstrained linear regression. The coefficient of determination for the 〈*v*_*x*_〉 data is very high, *r* ^2^=0.98, suggesting that the linear relationship between 〈*v*_*x*_〉 and *EF* provides a good explanation of the variability. In contrast, the coefficient of determination for 〈*v*_*y*_〉 is very low, *r* ^2^=0.00, suggesting that the null hypothesis is valid and there is no relationship between 〈*v*_*y*_〉 and *EF*. In summary, these results imply that a linear relationship between 〈*v*_*x*_〉 and *EF* is consistent with the observed data. To match the drift term in Eq. (1) with the advection-diffusion (Eq. (6)) we require that *v*_*x*_=*χ*(*S*)*∂* *S*/*∂* *x*. Since our data is consistent with a linear relationship between *v*_*x*_ and the applied gradient, *∂* *S*/*∂* *x*, it appears that a constant tactic sensitivity function, *χ*(*S*)=*χ*, provides the simplest explanation of our experimental results.

Results in Fig. 4 c-d show 〈*D*_*x*_〉 and 〈*D*_*y*_〉 as a function of *EF*. Visually, we see no discernible trend in the data for different values of *EF*. This visual interpretation is consistent with the fact that we obtain a small coefficient for each of the linear regressions in Fig. 4 c-d. Therefore, it is reasonable to assume that the cell diffusivities appear to be independent of the electric field. If we accept this assumption and further average the data in Fig. 3 i-p in each direction we obtain overall estimates of 〈*D*_*x*_〉=48*μ*m^2^/h and 〈*D*_*y*_〉=46*μ*m^2^/h. Again, this suggests that the diffusion of 3T3 fibroblast cells is approximately isotropic since we have *D*_*x*_≈*D*_*y*_, across all the experimental conditions considered.

Now that we have summarised the estimates of the directed and undirected components of cell migration in the experiments, we can quantify the relative roles in terms of the dimensionless Peclet number [38],

3 $$ Pe = \frac{v L}{D},  $$

where *v* is the drift velocity, *D* is the diffusivity and *L* is a relevant length-scale, which here we will take to be the cell diameter of fibroblast cells, *L*≈25*μ*m [37]. The Peclet number is a measure of the time scale of advection to the time scale of diffusion [38]. When *Pe*≪1, undirected diffusive transport dominates, when *Pe*≫1, directed transport dominates, and when *Pe*≈1 to two mechanisms are in balance. Comparing estimates of the drift velocity and the diffusivity in the *x*-direction suggests that our experiments deal with a range of Peclet numbers from *Pe*≈0 when *EF*=0 mV/mm to *Pe*≈10 when *EF*=400 mV/mm. Therefore, our experimental data covers a wide range of transport conditions ranging from purely undirected, diffusive transport to highly directed, advection-dominant conditions.

To summarise our findings, results in Fig. 4 suggest that 〈*v*_*x*_〉 increases linearly with *EF*, whereas the data suggests that the other transport coefficients, 〈*v*_*y*_〉, 〈*D*_*x*_〉 and 〈*D*_*y*_〉, appear to be independent of *EF*. Guided by these results, we assume that 〈*v*_*x*_〉 increases linearly with *EF*, and that the other transport coefficients are independent of *EF*. Comparing the results in Fig. 1 c and d also allows us to also consider whether there is any possible relationship between the transport coefficients and the electric potential. Repeating the process of plotting our estimates of the four transport coefficients as a function of the electric potential (not shown) suggests that there is no obvious trends in the data. Furthermore, linear regressions between each transport coefficient and the associated value of the electric potential reveals a low coefficient of determination, *r* ^2^<1. Therefore, based on the data, we assume that the transport coefficients appear to be independent of the electric potential in these experiments.

## Discussion

Our results indicate that when we quantify the roles of directed and undirected migration of 3T3 fibroblast cells under the influence of an applied electric field, the undirected component of the migration appears to be independent of the *EF*, and the directed migration appears to increase linearly with *EF*. Furthermore, we observe no consistent differences in the cell diffusivity estimates in the *x* and *y* Cartesian directions, implying that the undirected migration is isotropic. The simplest way to explain these results in terms of a Keller-Segel-type continuum model (Eq. (1)) is that we have a constant diffusivity, *D*(*S*)=*D*, and a constant chemotactic sensitivity function, *χ*(*S*)=*χ*. While the assumption that the chemotactic sensitivity function can be treated as a constant is widely invoked [24–33], this assumption is infrequently tested using experimental data collected under a range of gradient conditions. The question of whether the tactic sensitivity function ought to be treated as a constant or a more complicated expression is of interest because many theoretical models incorporate these kinds of details, such as receptor saturation, without necessarily being guided by experimental observations [6, 7, 13, 18–23].

## Conclusion

By examining trajectories of 3T3 fibroblast cells under a range of physiologically-relevant electric gradients [11, 14], we quantify the roles of directed and undirected migration. In summary we find that the undirected migration is isotropic and the cell diffusivity is approximately 50 *μ*m^2^/h, and that the drift velocity increases approximately linearly with the applied electric field, suggesting that the tactic sensitivity function is a constant.

Although our results apply to 3T3 fibroblast cells, we anticipate that repeating the experiments and analysis outlined here for different cell lines would provide insight into the roles of directed and undirected motility for any cell line of interest. Although we have found that the drift of fibroblasts to increase approximately linearly with the electric field in the range of *EF*=0−400 mV/mm, it is possible that we may observe a different response for different cell lines, or we may observe a different response for the same cell line when we apply a stronger electric field. However, here we deal only with gradients in the range of 0-400 mV/mm because this is a physiologically relevant range [11, 14].

## Methods

### Experimental methods

As shown in Fig. 1 a, we use a specifically designed and fabricated microfluidic chip to study the electrotaxis of NIH 3T3 fibroblasts. A CO_2_ laser scriber (ILS2, Laser Tools & Technics Corp, Taiwan) is used to ablate desired patterns on polymethylmethacrylate (PMMA) substrates [39–41]. Four layers of PMMA sheets are thermally bonded to form the fluidic channel, which is then attached to a cover glass to act as the cell culture area. The thickness of the fluidic channel is 1 mm, and the widths of the four culture areas (two copies) are 4.00, 8.28, 4.14 and 2.07 mm, respectively. By applying a direct current (dc) of 80 *μ*A, the *EF* inside these areas are calculated to be 0, 100, 200, and 400 mV/mm, respectively, based on Ohm’s law [35]. Numerical simulations of the *EF* and the potential inside the microfluidic chip is simulated using the commercial software package COMSOL Multiphysics (COMSOL, USA) to confirm these calculations (see Fig. 1 c and d).

The NIH 3T3 fibroblast cell line, purchased from Bioresource Collection and Research Center (BCRC, Taiwan), is cultured in a complete medium composed of Dulbecco’s modified Eagle medium (Gibco, USA) and 10% calf serum (Invitrogen, USA). 10^6^ cells are injected into the chip and the temperature is maintained at 37±0.5^o^C using a customized temperature controller. Different *EF* strengths are introduced by connecting the Ag(anode)/AgCl(cathode) electrodes (see Fig. 1 a) to a dc power supply (GWInstek, Taiwan) set at the constant-current model [42]. The microfluidic chip is mounted on a motorized, bright-field inverted microscope (CKX41, Olympus, USA) to observe cell migration. Figure 1 b shows an image of the cells in one culture area. For each culture area, corresponding to a different *EF*, images are taken over a period of 2 h. In each area, at least 80 cells were selected at random for data analysis.

### Modelling methods

Since we are dealing with trajectory data over a finite period of time for which no trajectory touches any physical boundary, we model the system as a random walk on *Ω*={(*x,y*):−*∞*<*x*<*∞*,−*∞*<*y*<*∞*}. For the analysis we denote the position of a cell at time *t*, relative to the position at *t*=0 as a random vector (*x*(*t*),*y*(*t*)), where *x*(*t*) and *y*(*t*) are the Cartesian coordinates at time *t*. These coordinates are related to a probability density function, *p*(*x,y,t*) so that

4 $$ \mathbb{P}\left\{ (x(t),y(t)) \in A \right\} = \iint_{A} p(x,y,t) \, \mathrm{d}x \, \mathrm{d}y,   $$

where *A* is a plane region that is a subset of *Ω*.

We take the simplest possible, standard approach by setting the transport coefficients, *D* and *χ*, to be constants [36, 43]. Furthermore, we also make use of the fact that the spatial gradient of electric field (*∂* *S*/*∂* *x*) is approximately constant across several subregions on the experimental device. However, at this stage we allow for the transport coefficients to potentially take on different values in different directions. These simplifications allow us to work with an anisotropic analogue of the linear advection-diffusion equation in a two-dimensional Cartesian geometry, which can be written as

5 $$ \frac{\partial{p}}{\partial{t}} = D_{x} \frac{\partial^{2} p}{\partial x^{2}}+D_{y} \frac{\partial^{2} p}{\partial y^{2}}- v_{x} \frac{\partial{p}}{\partial{x}} - v_{y} \frac{\partial{p}}{\partial{y}},  $$

on *Ω*. Since the distribution of cells on the experimental device is deliberately kept low so that the density of cells is well below carrying capacity, we deal with a linear model which is appropriate for cell migration under low cell density conditions where cell-to-cell collisions are relatively infrequent [44, 45]. If we consider the initial condition *p*(*x,y*,0)=*δ*(*x*)*δ*(*y*), which is relevant to following the motion of a single agent in the random walk starting from the origin [36], the solution of Eq. (5) is [43]

6 $$ {}p(x,y,t) = \frac{1}{4 \pi t \sqrt{D_{x} D_{y}}} \, \text{exp} \left[ \,-\, \left(\!\frac{(x-v_{x} t)^{2}}{4 D_{x} t} \,+\, \frac{(y-v_{y} t)^{2}}{4 D_{y} t}\!\right) \!\right].  $$

To interpret the random walk data in terms of this model we will deal with a series of individual trajectory data, (*x*(*t*),*y*(*t*)) with (*x*(0),*y*(0))=(0,0). We analyze each spatial component of the shifted trajectory separately. To achieve this we consider the marginal probability density functions for each spatial component,

$${}p_{x}(x,t) = \int_{-\infty}^{\infty} p(x,y,t) \, \mathrm{d}y, \quad \quad p_{y}(y,t) \,=\, \int_{-\infty}^{\infty} p(x,y,t) \, \mathrm{d}x, $$

and we evaluate the first two positive moments, the mean and variance, of *x*(*t*) and *y*(*t*), respectively. The first moments of the marginal probability density functions are given by

$${}\langle x^{1}(t) \rangle = \int_{-\infty}^{\infty} x \, p_{x}(x,t) \, \mathrm{d}x, \quad \quad \!\!\langle y^{1}(t) \rangle = \int_{-\infty}^{\infty} y \, p_{y}(y,t) \, \mathrm{d}y. $$

Using Eq. (6) we obtain

$$\langle x^{1}(t) \rangle = v_{x} t, \quad \quad \langle y^{1}(t) \rangle = v_{y} t. $$

Therefore, for each trajectory, (*x*(*t*),*y*(*t*)), we can obtain separate estimates of *v*_*x*_ and *v*_*y*_. Fitting a series of straight lines constrained to pass through the origin gives us an estimate of *v*_*x*_ and *v*_*y*_ for each trajectory. Since we have 80 trajectories for each gradient condition, we obtain 80 estimates of *v*_*x*_ and 80 estimates of *v*_*y*_. The variability amongst these estimates can be observed by plotting the results as a histogram. Furthermore, we can characterise the average coefficients by evaluating the sample mean and sample standard deviation of these 80 estimates. We will denote the sample mean as 〈*v*_*x*_〉 and 〈*v*_*y*_〉, respectively.

To provide information about the diffusivity, we will make use of the second moments of the marginal probability density functions are given by

$$\begin{aligned} \left\langle x^{2}(t) \right\rangle &= \int_{-\infty}^{\infty} \left(x - \left\langle x^{1}(t)\right\rangle\right)^{2} \, p_{x}(x,t) \, \mathrm{d}x,\\ &\quad \times\left\langle y^{2}(t) \right\rangle = \int_{-\infty}^{\infty} \left(y - \left\langle y^{1}(t)\right\rangle\right)^{2} \, p_{y}(y,t) \, \mathrm{d}y. \end{aligned} $$

Using Eq. (6) we obtain

$$\left\langle x^{2}(t)\right\rangle = 2 D_{x} t, \quad \quad \left\langle y^{2}(t) \right\rangle = 2 D_{y} t. $$

Therefore, given our previous estimates of the average drift velocity in each direction 〈*v*_*x*_〉 and 〈*v*_*y*_〉, for each trajectory we can obtain separate estimates of *D*_*x*_ and *D*_*y*_. Fitting a series of straight lines constrained to pass through the origin give us estimates of *D*_*x*_ and *D*_*y*_ for each trajectory. Since we have 80 trajectories for each gradient condition, we obtain 80 estimates of *D*_*x*_ and 80 estimates of *D*_*y*_. The variability amongst these estimates can be observed by plotting the results as a histogram. Furthermore, we can characterise the average coefficients by evaluating the sample mean and sample standard deviation of these 80 estimates. We will denote the sample mean as 〈*D*_*x*_〉 and 〈*D*_*y*_〉, respectively.

## Additional file

Additional file 1 Additional data. Numerical estimates of transport coefficients. (XLSX 21 kb)

## Abbreviation

EF: electric field
